# Visualised genotyping assay with oral swabs in a closed tube by nested invasive reaction assisted with gold nanoparticle probes

**DOI:** 10.1049/nbt2.12123

**Published:** 2023-03-10

**Authors:** Yijun Li, Wei Wei, Yi Ma, Jingwen Shan, Yanan Chu, LiKun Zhang, Danni Liu, Xueping Ma, Guohua Zhou, Haiping Wu

**Affiliations:** ^1^ Department of Clinical Pharmacy Jinling Hospital School of Pharmaceutical Sciences Southern Medical University Guangzhou China; ^2^ State Key Laboratory of Analytical Chemistry for Life Science and Jiangsu Key Laboratory of Molecular Medicine Medical School of Nanjing University Nanjing China; ^3^ School of Life Science and Technology China Pharmaceutical University Nanjing China; ^4^ School of Pharmacy Nanjing Medical University Nanjing China

**Keywords:** nanobiotechnology, nanoparticles, patient treatment

## Abstract

Single nucleotide polymorphism (SNP) typing is crucial for drug dosage and disease progression. Therefore, a simple and convenient genotyping assay is essential for personalised medicine. Herein, we developed a non‐invasive, closed‐tube, and visualised method for genotyping. In this method, oral swabs were lysed to directly perform PCR coupled with nested invasive reaction and visualisation based on gold nanoparticle probes in a closed tube. The strategy for genotyping assay depends on the single base recognition property of invasive reaction. This assay allowed quick and simple sample preparation and the detection of 25 copies/μL of CYP2C19*2 and 100 copies/μL of CYP2C19*3 within 90 min. Further, 20 oral swab samples for CYP2C19*2 and CYP2C19*3 were correctly typed, which agreed with pyrosequencing, indicating that this method has great potential for SNP typing in source‐limited regions to guide personalised medicine.

## Graphical abstract

A non‐invasive, closed‐tube and visualised genotyping assay with oral swabs to guide individualised therapy.

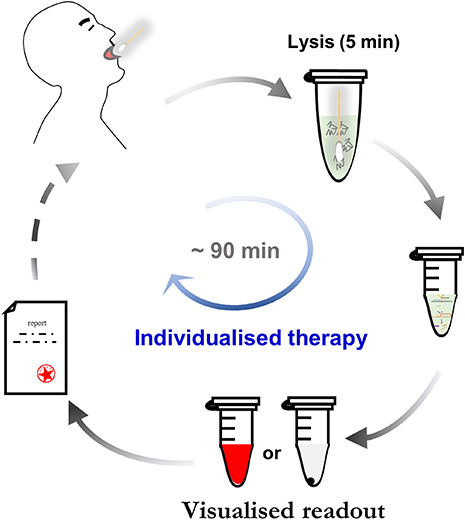

## 1 INTRODUCTION

Single nucleotide polymorphism (SNP) refers to DNA sequence polymorphism caused by single nucleotide variation at the genomic level [1, 2]. SNP is related to the development of many human diseases and individual drug responses [3, 4]. The polymorphism of cytochrome P450 genes affects therapeutic outcomes and adverse reactions of drugs that are metabolised by the cytochrome P450 enzyme [5, 6]. For example, Genetic polymorphisms on CYP2C19*2 and CYP2C19*3 affect the efficacy and adverse effects of clopidogrel [7], as well as the clinical prognosis of patients [8, 9]. Therefore, the relevant SNP is suggested to detect before the medication of such drugs [10]. Consequently, developing convenient and sensitive genotyping methods is of great clinical significance.

Commonly, methods for SNP typing are mainly based on the amplification of nucleic acid coupled with the detection of amplicons. Polymerase chain reaction (PCR) is a classical method of nucleic acid amplification, and recently emerging isothermal amplification technology such as recombinase polymerase amplification (RPA) [11, 12] and loop‐mediated isothermal amplification (LAMP) [13, 14] is also increasingly used for nucleic acid amplification. Sequencing [15, 16] and fluorescence‐based methods [17, 18, 19, 20, 21, 22], such as molecular beacons, TaqMan probes, and CRISPR/Cas systems, are usually used for the detection of amplificons. However, these methods require either expensive reagents or instruments to sequence or read out fluorescence signals. And most of them need to transfer the amplified products to the detection steps, increasing the operation steps and the risk of amplicons contamination. Therefore, these methods have limitations for large‐scale applications. In addition, due to the requirement for high‐quality samples, most of these methods usually need genome DNA (gDNA) extracted and purified from blood samples [23], which is a time‐consuming and labour‐intensive sample preparation method that also brings discomfort to patients.

Thus, a simple and cost‐effective detection method needs to be developed to provide better treatment for patients, especially in limited‐resource areas. Oral swabs, as non‐invasive samples, are increasingly being used for disease diagnosis [24, 25]. Based on our previous work [26], here, we established a non‐invasive, closed‐tube and visualised genotyping assay using PCR coupled with invasive reaction assisted with gold nanoparticle probes hybridisation using oral swab samples lysed with a simple method. We eliminate complex steps for purifying the sample, and the whole genotyping method can be completed only with a PCR engine, which is non‐invasive, cost‐effective, and convenient for application in limited‐resources areas.

## 2 MATERIALS AND METHODS

### 2.1 Reagents and equipment

Reagents included dNTPs (SBS Genetech Co., Ltd., Shanghai, China), GoTaq Hot Start polymerase (Promega, Beijing, China), PEG8000(BSK Technology Co., Ltd., Nanjing China) and mineral oil (Sigma‐Aldrich, St. Louis, MO). Tris, MgCl_2_·6H_2_O, and NaCl were purchased from Sinopharm Chemical Reagent Co., Ltd. (Shanghai, China). Nonidet P‐40 and Tween‐20 were obtained from Amresco. A 10× reaction buffer (10× IB buffer) containing 100 mM Tris−HCl (pH 8.5), 300 mM NaCl, 75 mM MgCl_2_, 0.5% Nonidet P‐40, and 0.5% Tween‐20 was prepared in our laboratory. Reagents of gold nanoparticle probes (AuNP‐probes) preparation included (3‐Mercaptopropyl)‐trimethoxy silane (Sigma‐Aldrich LLC), Chloroauric acid tetrahydrate (Sinopharm Chemical Reagent Co., Ltd., Shanghai, China), and sodium citrate (Sinopharm Chemical Reagent Co., Ltd., Shanghai, China). Flap endonuclease 1 (FEN1) and AuNP‐probes were prepared in our laboratory as published procedure [27, 28, 29]. Lysis solution for oral swabs included Lysis Buffer (Yaneng bio, Shenzhen China), QuickExtract™ (Lucigen, Beijing China) and Tris‐HCl Buffer containing 67 mM Tris‐HCl. Equipment included a PCR machine (A200 Gradient Thermal Cycler, Long Gene) and One Drop OD‐1000 (Wuyi Technology Co., Ltd., Nanjing, China).

### 2.2 Primers and probes design

Sequences information of different genotypes of CYP2C19*2 (G681 A, rs17879456), CYP2C19*3 (G636A, rs4986893), CYP2C9*2 (C430T, rs1799853), CYP2C9*3 (A1075C, rs1057910) were derived from the NCBI, and plasmid templates were synthesised by Invitrogen (Shanghai, China). PCR primers were designed by Primer 5.0 software. The upstream probes (UPs) and downstream probes (DPs) were designed by Universal Invader™ Software. All PCR primers and probes were synthesised by Sangon (Shanghai, China). The sequence information of primers and probes were listed in Table S1.

### 2.3 Preparation of silica‐modified AuNP‐probes

Firstly, the gold nanoparticles (AuNPs) with an average of 13 nm were synthesised by reduction of sodium citrate and chloroauric acid. Secondly, the AuNPs were modified by two oligonucleotide probes complementary to the 5′ end and 3′end of the hairpin probe, respectively. Then the AuNP‐probes were modified with (3‐Mercaptopropyl)‐trimethoxy silane to build a protective layer which could prevent AuNPs from absorbing enzymes and modified probes from falling off the surface of AuNPs at high temperatures. The AuNPs were characterised by transmission electron microscopy (TEM) (Figure S1a), and AuNP‐probes and silica‐modified AuNP‐probes were characterised by UV‐visible spectrophotometer (Figure S1b).

### 2.4 Sample collection and processing

Genomic DNA (gDNA) from blood was extracted and purified by the DNA extraction kit (Progema, A1125). The heads of oral swabs were broken off and inserted into 250 μL of different lysis solutions from different lysis methods, respectively. Then the solution was processed, respectively, by different lysis methods. Next, the head of the oral swab was squeezed and removed. The lysate was centrifuged at 12,000 g for 30 s, and the supernatant was collected for direct visualised genotyping assay. The lysis methods to be selected contained mechanical cracking method: ultrasound (Method A, the head of oral swab was immersed into 250 μL of H_2_O and incubated for 5 min in an ultrasonic bath), and three chemical cracking methods: Lysis Buffer (Method B, the head of oral swab was immersed into 250 μL of Lysis Buffer and heated at 100°C 10 min), QuickExtract^TM^ (Method C, the head of oral swab was immersed into 250 μL of QuickExtract^TM^ solution and heated at 65°C for 6 min and 98°C for 30 s), and Tris‐HCl Buffer(Method D, the oral swab head was immersed into 250 μL of Tris‐HCl Buffer and heated at 96°C for 5 min). All samples were collected from Jinling Hospital (Nanjing, China).

### 2.5 Visualised genotyping assay

The visualised genotyping was performed in a 20‐μL reaction system containing 2 μL of 10× IB buffer, 0.25 mM dNTPs, 500 nM forward primer, 500 nM reverse primer, 0.25 U Go Taq DNA polymerase, 3.5% PEG8000, 100 nM UP, 200 nM DP, 200 nM hairpin probe, 40 ng of FEN1, 2 μL of each AuNP‐probes (AuNP‐1 and AuNP‐2, Table S1), and 2 μL of samples. Then the reaction was performed as follows: 94°C for 1 min, 35 cycles of (94°C for 5 s and 72°C for 40 s), 72°C for 2 min, 94°C for 5 s, 63°C for 10 min, 55°C for 20 min, and 4°C for 1 min. Finally, the reaction tubes were centrifuged in a mini‐centrifuge for 30 s. The result could be observed by the naked eyes, with positive tubes in red and negative tubes in colourless after centrifugation.

### 2.6 Sensitivity and specificity

To evaluate the sensitivity of visualised genotyping assay under the lysis buffer of our selected method, human gDNA purified from blood was added into lysis buffer to prepare the simulated samples of oral swab lysate. And 2‐μL simulated samples of heterozygotes of different concentrations (1, 10, 25, 50, 100 and 1000 copies/μL) were added into 18 μL of the reaction system, respectively. To investigate the specificity, gDNA at about 10^4^ copies/μL of allele‐G and allele‐A was crossly added into the system for typing allele‐G and allele‐A.

### 2.7 SNP typing of clinical samples

To validate the visualised genotyping assay, 20 oral swab samples from Jingling hospital were analysed by our proposed method for genotyping of CYP2C19*2 and CYP2C19*3. Oral swabs were directly lysed by a commercial lysis buffer (Yaneng bio) to release gDNA. And the 20 oral swabs were also tested by pyrosequencing.

## 3 RESULTS

### 3.1 Principle

**FIGURE 1:**
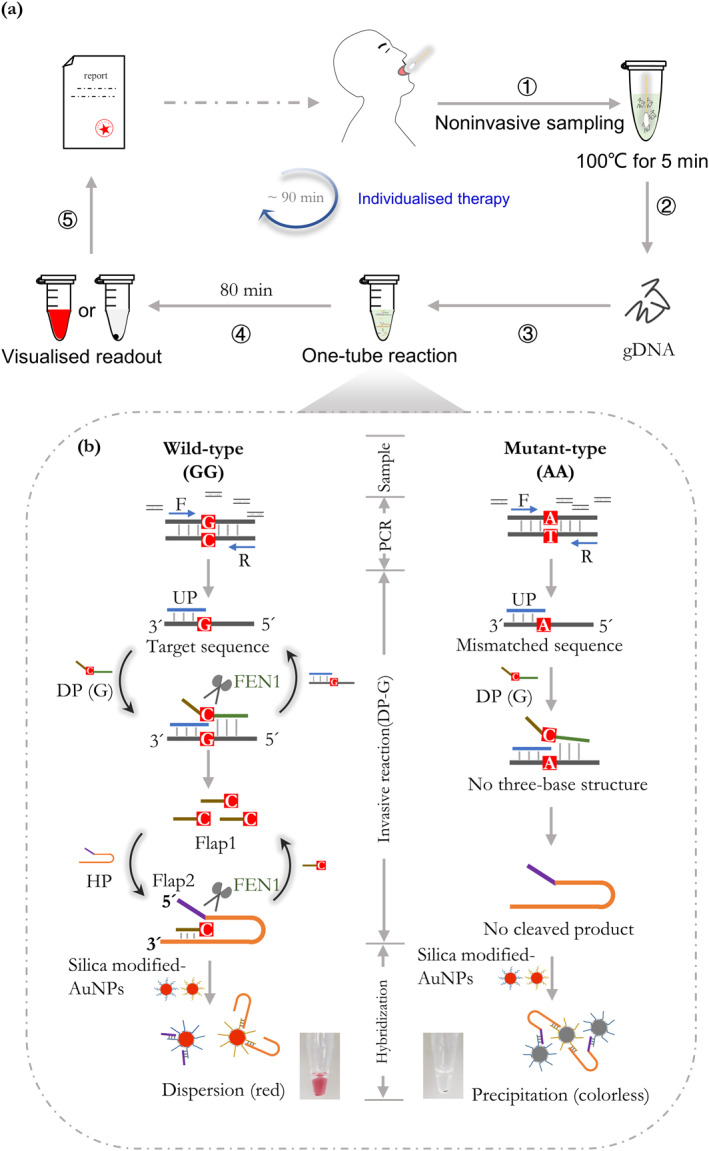
The scheme of closed‐tube visualised genotyping assay. (a) Workflow of the whole genotyping assay. (b) Principle of the one‐tube reaction by PCR coupled with nested invasive reaction assisted with AuNP‐probes.

The workflow of our proposed method is shown in Figure 1, including four sections, which are: oral swabs lysis, PCR, invasive reaction, and AuNP‐probes hybridisation (Figure 1a). Firstly, oral swabs were collected and simply heated at 100°C for 5 min to release gDNA. Then, the visualised genotyping assay was performed in a closed tube and the detection result could be observed by the naked eyes. Finally, the genotyping report was given to guide the individualised medication of patients in time.

The principle of the one‐tube reaction was shown in Figure 1b. This was clarified using a representative SNP (CYP2C19*2). Two possible bases, either G or A, may be located at this polymorphic site. The target containing the SNP sites of interest was amplified by PCR, and then the SNP type was detected by the invasive reaction. Take DP for typing allele‐G as an example, when the DP with base‐C (DP for typing allele‐G) was hybrid with the template of allele‐G, the UP, DP and template formed a three‐base structure that could be recognised by the FEN1. Then the Flap1 fragment of DP was cleaved by activated FEN1, and the Flap1 fragment could invade the hairpin probe (HP) forming a three‐base structure. The HP was also cleaved into two parts (Flap2 and residual HP) by activated FEN1. Finally, two kinds of AuNP‐probes could be complementary to the Flap2 and residual HP, respectively, and dispersed in the tube in red. On the other hand, when the DP for typing allele‐G and the template of allele‐A met, the invasive reaction would not occur, and the HP was kept complete. The two kinds of AuNP‐probes were hybridised by the hairpin probe and deposited at the bottom of the tube after centrifugation.

Systems of DP for typing allele‐G and for typing allele‐A would be prepared for one sample to investigate whether the samples contained allele‐G or A.

### 3.2 Optimisation for one‐tube reaction

**FIGURE 2:**
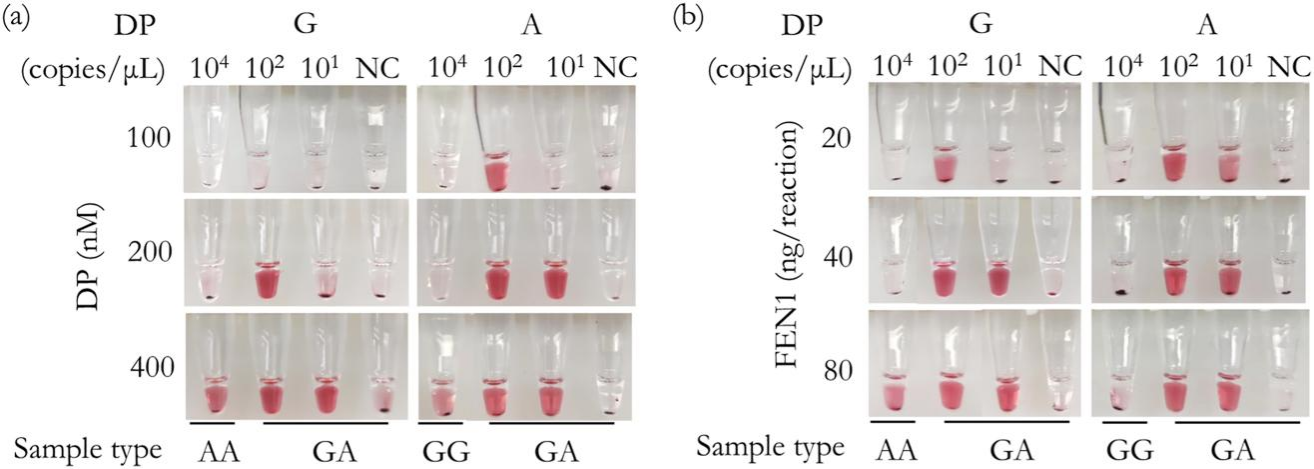
Optimization of the (a) DP concentrations and the (b) FEN1 concentrations by detecting CYP2C19*2. “NC” is the control without any target.

DP probes and FEN1 play a key role in the specificity of the invasive reaction, because the background will appear with the concentration increasing. To get good specificity without any loss in sensitivity, different DP concentrations (400, 200, and 100 nM) and different FEN1 concentrations (80, 40, 20 ng/reaction) were investigated. As shown in Figure 2, the optimised DP concentration and FEN1 amount in the invasive reaction for producing specific signals were 200 nM and 40 ng/reaction, respectively.

**FIGURE 3:**
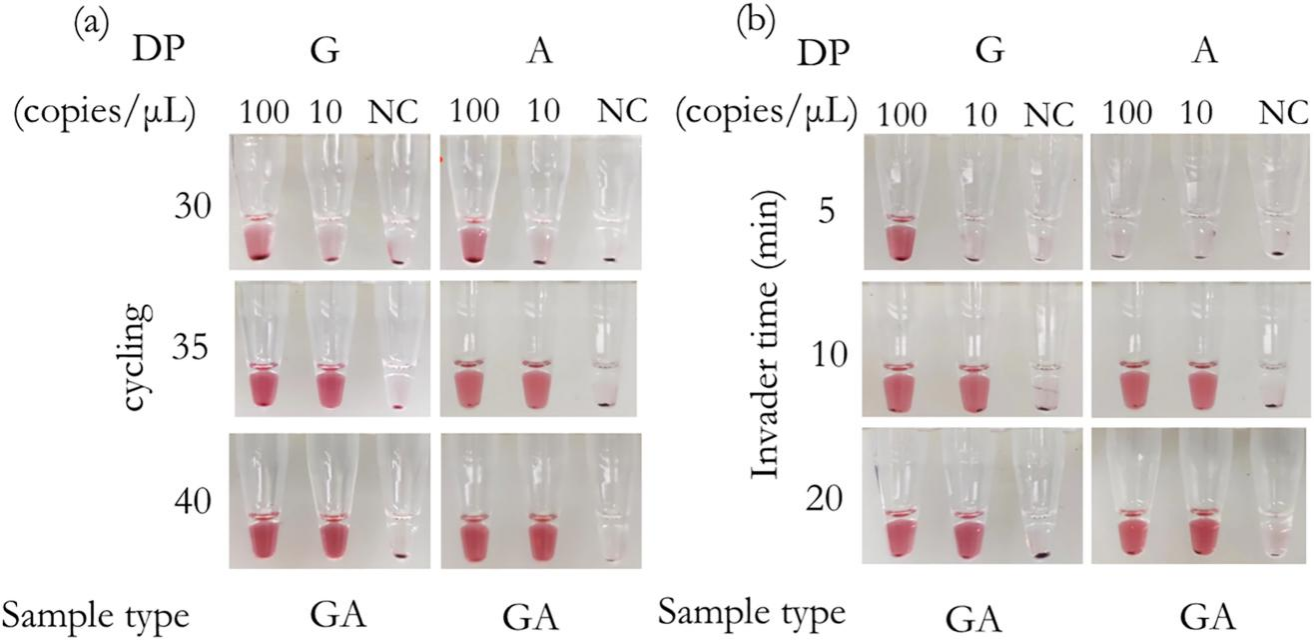
Optimization of the (a) PCR cycling numbers and the (b) invasive reaction time by detecting CYP2C19*2. “NC” is the control without any target.

Furthermore, we optimised the PCR cycling numbers and invasive reaction time, as they were important for sensitivity. As shown in Figure 3, with the increase of the PCR cycling numbers or the invasive reaction time, the sensitivity became better. But a shorter reaction time is more advantageous for detection at the same sensitivity. Therefore, we chose an invasive reaction time of 10 min and PCR cycling numbers of 35 as optimal conditions.

### 3.3 Selection and optimisation of lysis method of oral swabs

**FIGURE 4:**
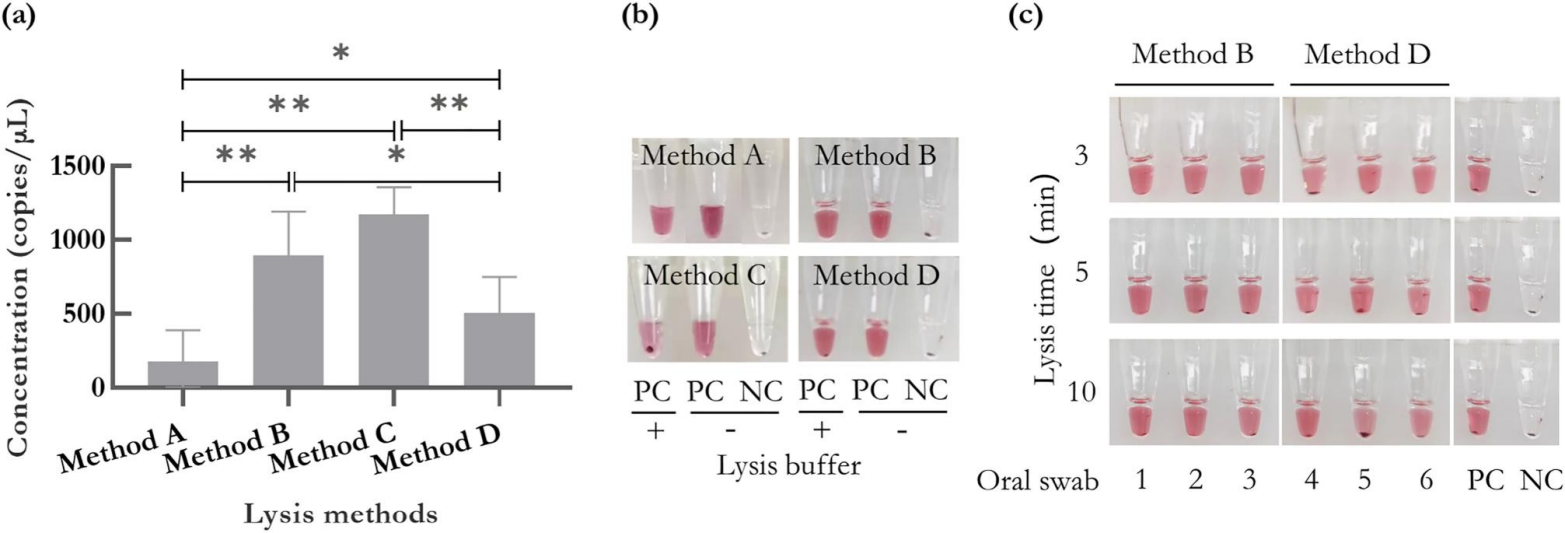
(a) The average concentrations of gDNA lysed from 6 oral swabs by four kinds of methods (177 ± 212, 893 ± 297, 1174 ± 184 and 503 ± 245 copies/μL, respectively). (b) Effect of buffers used in the four methods on the visualised genotyping assay. (c) Images of the visualised genotyping assay on three oral swabs individually lysed by Methods B and D for 3, 5 and 10 min, respectively. “PC” is the control with 200 copies of gDNA. “NC” is the control without any target. “*” means *P* < 0.05, “**” means *P* < 0.01.

To lyse oral swabs and perform visualised genotyping reactions quickly, it is necessary to select a fast lysis method that does not affect the visualised reaction. Four methods (Methods A, B, C, and D) described in MATERIALS AND METHODS lysed six oral swabs, respectively, and gDNA concentration was quantified. The average concentrations of gDNA obtained by four methods were 177 ± 212, 893 ± 297, 1174 ± 184 and 503 ± 245 copies/μL, respectively (Figure 4a). In addition to Method A, the other three methods could obtain sufficient gDNA from oral swabs. Next, we explored the effect of lysis buffers on the visualisation reaction. 2 μL of buffers and 200 copies of gDNA were contained with a 20‐μL reaction system. In this experiment, we set the positive control with a gDNA of 200 copies, which can be stably detected. We observed that the buffer of Method C could cause weak signal for the visualised detection (Figure 4b). Considering both the efficiency of lysis and effect on visualised detection, we chose methods B and D for further investigation.

Next, we explored the effect of crude cell lysates prepared by Methods B and D through visualised detection. Crude cell lysates of three oral swabs, suffered from the lysis of 3, 5 and 10 min, respectively, were detected. Unexpectedly, weak signal appeared randomly at each lysis time when using method D for lysis (Figure 4c), implying that method D is not efficient to remove complex biological substrates such as lysed human cells and mucosal secretions in swabs after centrifugation. Consequently, method B is the best way for cell lysis when coupled with the proposed visualised genotyping detection.

**FIGURE 5:**
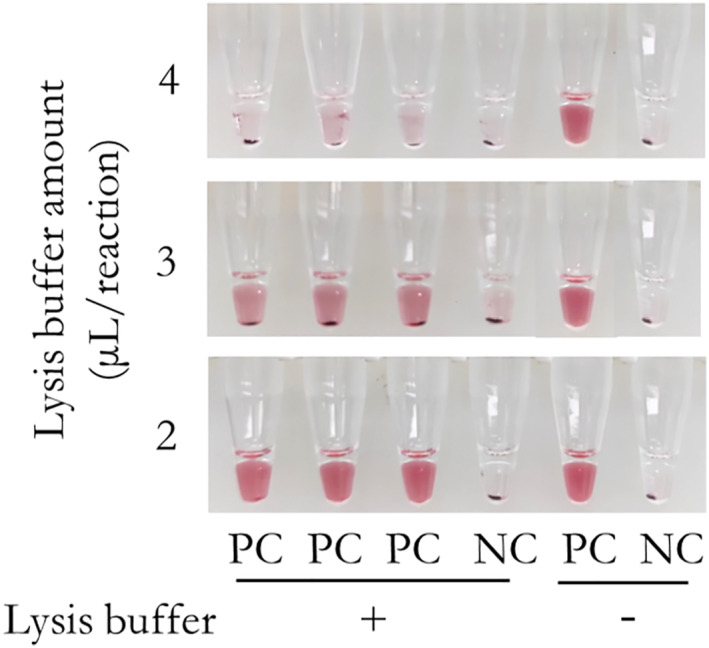
Effect of the concentrations of lysis buffer of Method B on visualised genotyping assay of CYP2C9*3. “PC” is the control with 200 copies of gDNA. “NC” is the control without any target.

**FIGURE 6:**
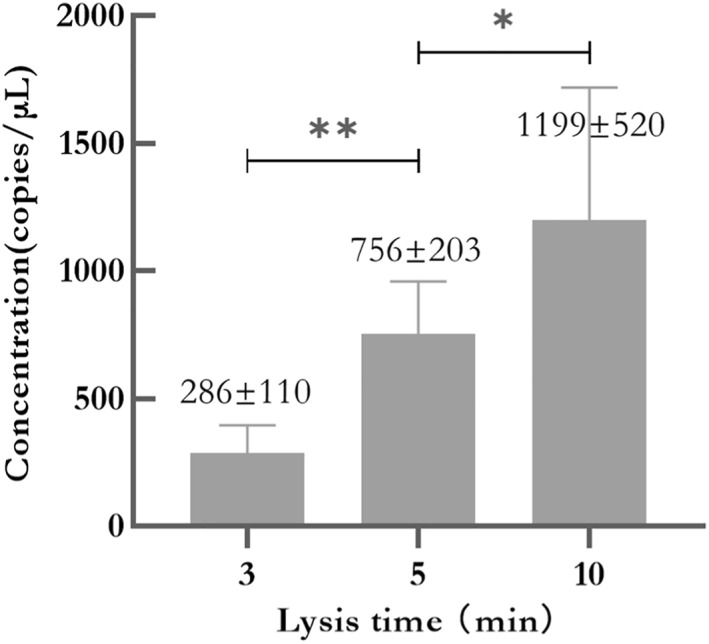
The average concentrations of gDNA lysed by Method B from nine oral swabs for 3, 5, and 10 min “*” means *P* < 0.05, “**” means *P* < 0.01.

Furthermore, with an increase in the volume of lysis buffer of method B, there was a corresponding decrease in positive signals (Figure 5). Based on the results, 2 μL of the sample was found suitable for direct visualised detection. In addition, according to the instruction, method B for lysis is recommended to be 10 min. But the results in Figure 4c also suggested that the lysate of oral swabs for different lysis time by method B could be well detected, which provided a reference for our next lysis time optimisation. For fast detection, we optimised the lysis time. Crude cell lysates from 9 oral swabs for lysis times of 3, 5 and 10 min were quantitated, and the average concentrations of gDNA obtained from oral swabs were 286 ± 110, 756 ± 203 and 1198 ± 520 copies/μL (Figure 6), respectively. To ensure sufficient templates and a shorter lysis time, we finally chose the lysis time of 5 min.

### 3.4 Sensitivity and specificity

**FIGURE 7:**
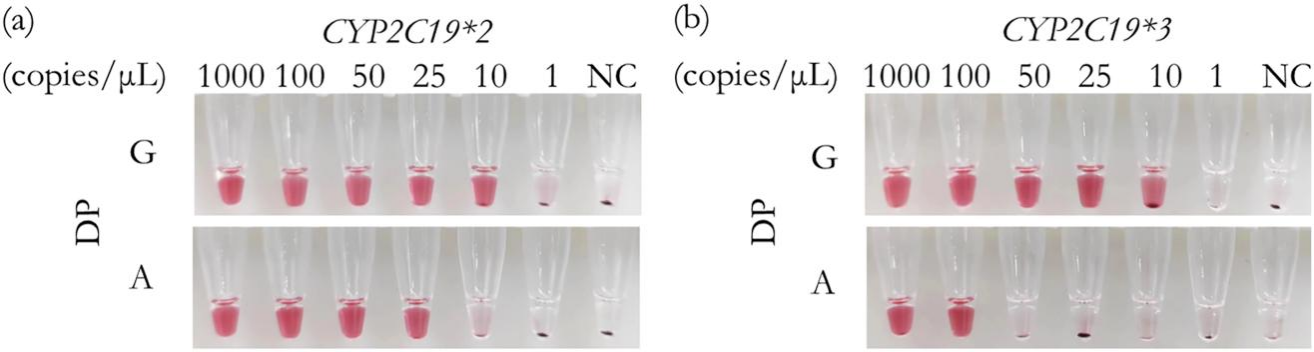
Sensitivity of the visualised genotyping assay on (a) CYP2C19*2, (b) CYP2C19*3. “NC” is the control without any target.

The sensitivity of the visualised genotyping assay under the lysis buffer was investigated. Simulated clinical samples of a series of concentration were used to detect CYP2C19*2 and CYP2C19*3. As shown in Figure 7, the limit of detection of our methods was 25 copies/μL of CYP2C19*2 and 100 copies/μL of CYP2C19*3, which could meet the needs of oral swab detection. The reason of the little higher sensitivity of allele‐G than allele‐A for both CYP2C19*2 (G/A) and CYP2C19*3 (G/A) is believed to be from the slight increase of the melting temperature in the downstream probes used for typing allele G. As shown in the mechanism illustrated in Figure 1, the signal produced by the FEN1‐catalysed cleavage reaction is dependent on the dynamic process in the hybrids formed by the target and downstream probes. Once the downstream probe is cleaved, the intact probe should replace the cleaved one and hybridise with the target immediately, but this process needs the temperature very close to the Tm of the probes. Since there is 0.5°C difference in Tm between the probe with base‐G and the probe with base‐A, the probes for typing allele‐G in CYP2C19*2 and *3 are easier to capture the corresponding target at a given temperature, accordingly causing the inconsistent sensitivity for two alleles as indicated in Figure 7.

**FIGURE 8:**
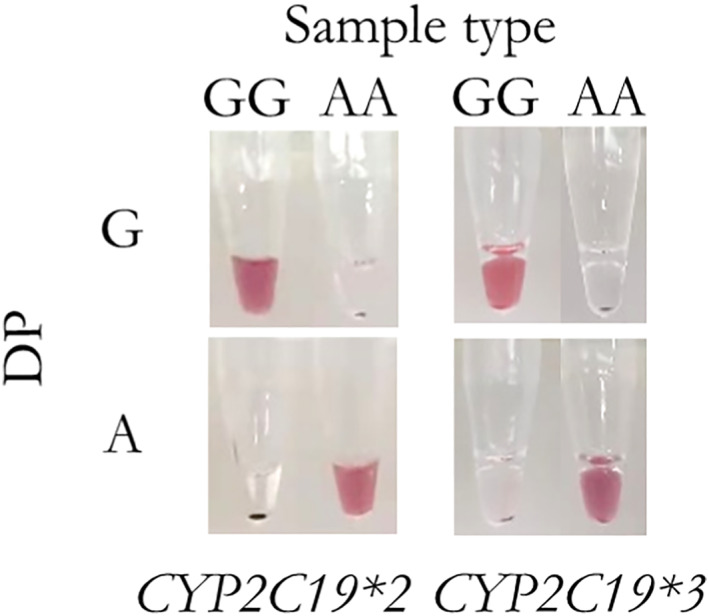
Specificity of visualised genotyping assay on CYP2C19*2 and CYP2C19*3.

Meanwhile, it is important to investigate the specificity of our methods for accurate genotyping. We mixed different types of probes and templates to investigate the non‐specific signals of both CYP2C19*2 and CYP2C19*3. As shown in Figure 8, there was no background of the genotyping of both CYP2C19*2 and CYP2C19*3. Thus, our methods could well identify the single base variation.

### 3.5 Sample testing

**FIGURE 9:**
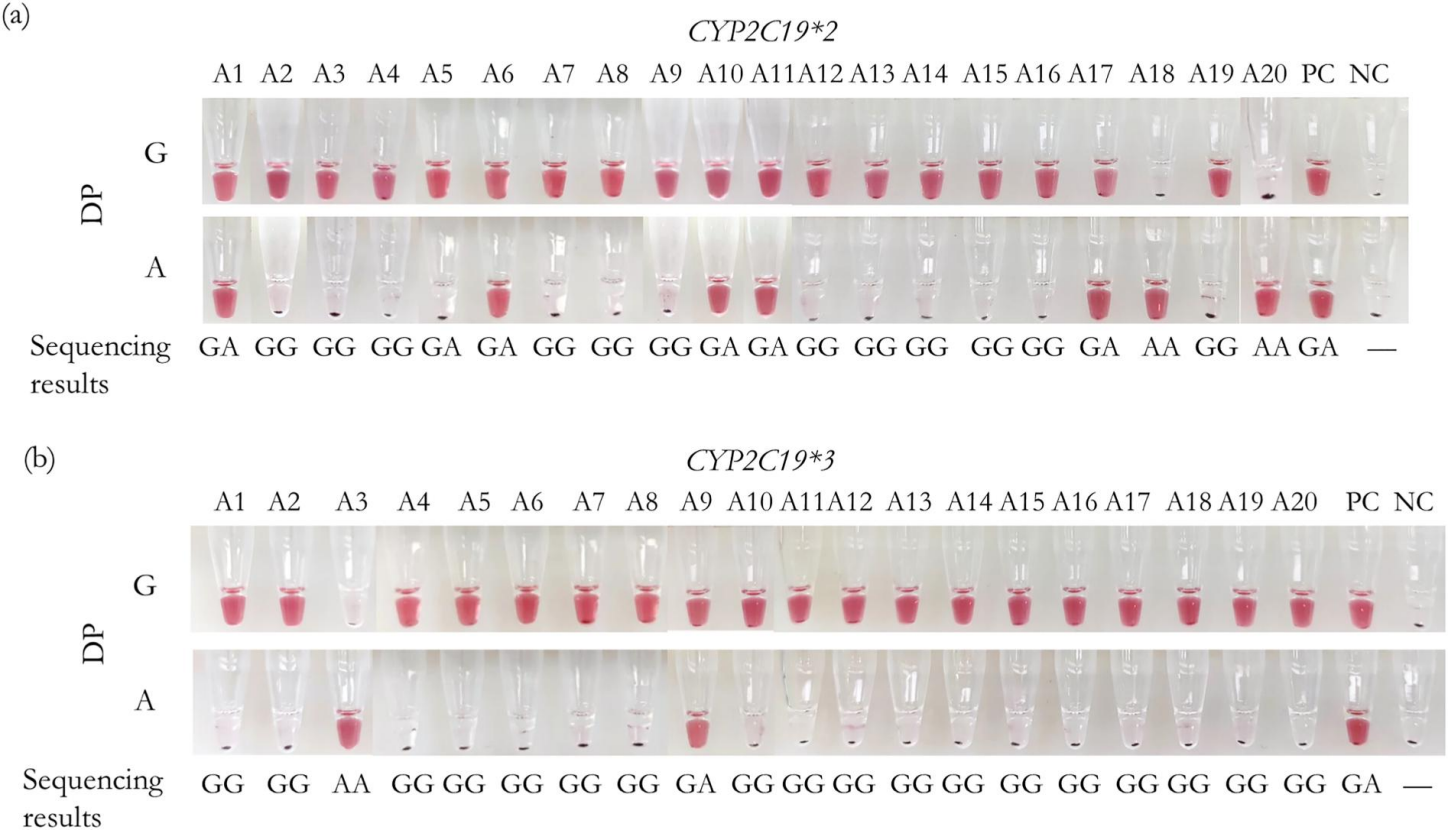
Images of the visualised genotyping with 20 real‐world oral swab samples by targeting (a) CYP2C19*2 and (b) CYP2C19*3 of clopidogrel individualised medication‐related sites, respectively. The genotype of each sample by pyrosequencing is listed at the bottom of each tube. A1∼A21 are samples of oral swabs. “PC” is the control with 200 copies of gDNA. “NC” is the control without any target.

To validate the ability of the visualised genotyping assay for detecting real samples, we collected 20 clinical samples for the detection of CYP2C19*2 and CYP2C19*3, which related to individualised treatment of clopidogrel, including 13 wild homozygotes, 5 heterozygotes and 2 mutant homozygotes of CYP2C19*2 and 18 wild homozygotes, 1 heterozygote and 1 mutant homozygotes of CYP2C19*3. As shown in Figure 9, red signals were observed for samples with the corresponding genotype, while those without the corresponding genotype were colourless. And all these results were consistent with pyrosequencing (Table S2), indicating that our method is of high accuracy to be used for personalised medicine‐related gene detection in resource‐constrained areas.

## 4 CONCLUSIONS

With the development of visualised detection technology for molecular diagnosis, increasingly visualised methods are used for SNP detection, such as lateral flow dipstick [30], pH‐sensitive dyes [31], and enzyme‐linked immunosorbent assay [14]. These methods require open‐tube operation with a high risk of cross‐contamination. Our previous work has successfully formed SNP discrimination based on AuNP‐probes [26, 32], but gDNA still needed to be purified from blood, which inevitably increases patient suffering.

Here, we proposed a closed‐tube visualised method for genotyping with rapid lysis of oral swabs and validated our method by genotyping the clopidogrel SNPs. Samples can be prepared within 6 min, and the whole turnaround time is within 90 min. This is a cost‐effective method that requires only a simple PCR engine, and all reactions can be carried out sequentially in a tube, reducing cross‐contamination of the amplicons. Our method does not bring discomfort to patients so it can improve compliance of patients, thus very suitable for the detection of individualised therapy‐related genes in limited‐resources areas. And the visualised genotyping assay could be widely used, such as typing other SNPs and in vitro diagnosis or prognosis of other disease‐related genes in clinical settings.

## AUTHOR CONTRIBUTIONS

**Yijun Li**: Data curation, Methodology, Validation, Visualisation, Writing – original draft. **Wei Wei**: Writing – review & editing. **Yi Ma**: Writing – review & editing. **Jingwen Shan**: Writing – review & editing. **Yanan Chu**: Supervision. **Likun Zhang**: Methodology. **Danni Liu**: Writing – review & editing. **Xueping Ma**: Supervision. **Guohua Zhou**: Conceptualisation, Resources, Writing – review & editing. **Haiping Wu**: Project administration, Writing – review & editing.

## CONFLICT OF INTEREST STATEMENT

The authors do not have any possible conflicts of interest.

## Supporting information

Supporting Information S1

## Data Availability

The data supporting this study's findings are available from the corresponding author upon reasonable request.
