# Supplementary material for: Visualised genotyping assay with oral swabs in a closed tube by nested invasive reaction assisted with gold nanoparticle probes
Source: IET Nanobiotechnol. 2023 Mar 10;17(3):281–8. doi: 10.1049/nbt2.12123 (PMC10190604; doi:10.1049/nbt2.12123)
Supplement: Supplementary file 1 — Supporting Information S1 [file NBT2-17-281-s001.docx]

Supporting Information

Visualized genotyping assay with oral swabs in a closed tube by nested invasive reaction assisted with gold nanoparticle probes

Yijun Li^1, †^, Wei Wei^1, †^, Yi Ma^3^, Jingwen Shan^1^, Yanan Chu^1^, LiKun Zhang^3^, Danni Liu ^2^, Xueping Ma ^1^, Guohua Zhou^1,2,3,4,*^, and Haiping Wu^1,2,*^

1 Department of Clinical Pharmacy, Jinling Hospital, School of Pharmaceutical Sciences, Southern Medical University, Guangzhou, 510515, China

2 School of Life Science and Technology, China Pharmaceutical University, Nanjing, 210009, China

3 State Key Laboratory of Analytical Chemistry for Life Science & Jiangsu Key Laboratory of Molecular Medicine, Medical School of Nanjing University, Nanjing, 210023, China

4 School of Pharmacy, Nanjing Medical University, Nanjing, 211166, China

† Yijun Li and Wei Wei contributed equally to this work.

***Correspondence:**

Haiping Wu, Department of Clinical Pharmacy, Jinling Hospital, School of Pharmaceutical Sciences, Southern Medical University, Guangzhou, 510515, China.

Email: wuhaiping@smu.edu.cn

Guohua Zhou, Department of Clinical Pharmacy, Jinling Hospital, School of Pharmaceutical Sciences, Southern Medical University, Guangzhou, 510515, China.

Table of Contents

| Supporting figure 1 | Characterization of AuNPs, AuNP-probes and Silica-Modified AuNP-probes. | S4 |
| --- | --- | --- |
| Supporting Table 1 | Sequences of primers and probes. | S5 |
| Supporting Table 2 | Pyrosequencing map of Samples. | S6 |

Supporting figure 1. Characterization of AuNPs, AuNP-probes and Silica-Modified AuNP-probes.


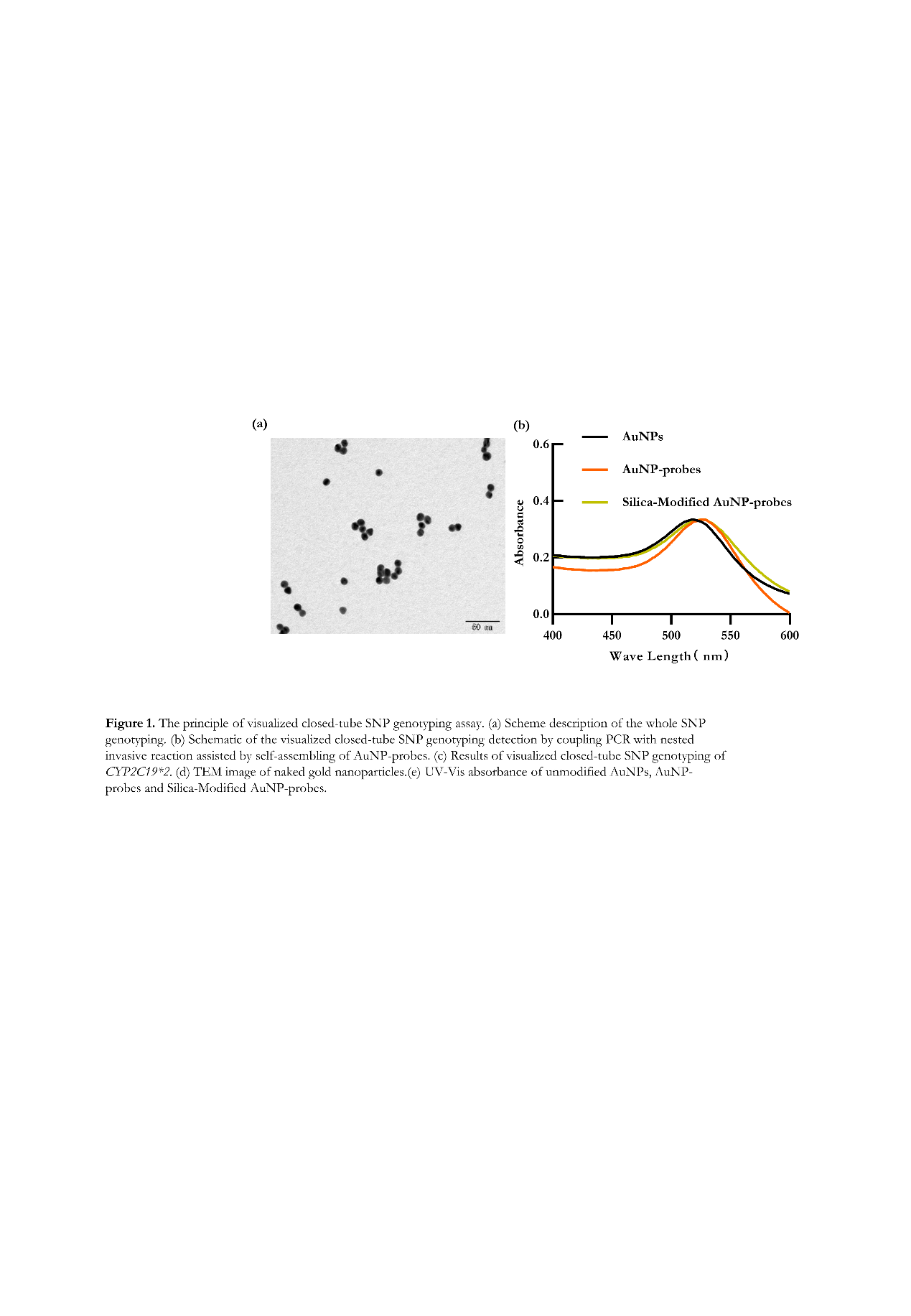


Figure S1: (a) TEM image of AuNPs. (b) UV-Visible absorbance of AuNPs, AuNP-probes and Silica-Modified AuNP-probes.

Supporting Table 1. Sequences of primers and probes.

| primers and probes | Sequence (5´-3´) |
| --- | --- |
| *CYP2C19*2*-F | CCAGAGCTTGGCATATTGTATCTATACCTTTATTAAATGC |
| *CYP2C19*2*-R | CCATCGATTCTTGGTGTTCTTTTACTTTCTCCAA |
| *CYP2C19*2*-UP | CAAGGTTTTTAAGTAATTTGTTATGGGTTCCA |
| *CYP2C19*2*-DP-W | *CGCGCCGAGG* **C**GGGAAATAATCAATGATAGTG-C_3_ |
| *CYP2C19*2*-DP-M | *CGCGCCGAGG* **T**GGGAAATAATCAATGATAGTG-C_3_ |
| *CYP2C19*3*-F | TCTGCTCCATTATTTTCCAGAAACGTTTCGA |
| *CYP2C19*3*-R | ACTGTAAGTGGTTTCTCAGGAAGCAAAAAACT |
| *CYP2C19*3*-UP | AGGATTGTAAGCACCCCCTGT |
| *CYP2C19*3*-DP-W | *CGCGCCGAGG* **G**ATCCAGGTAAGGCCA-C_3_ |
| *CYP2C19*3*-DP-M | *CGCGCCGAGG* **A**ATCCAGGTAAGGCCA-C_3_ |
| *CYP2C9*2*-F | TGACGCTGCGGAATTTTGGGAT |
| *CYP2C9*2*-R | CTCCTCCACAAGGCAGCGG |
| *CYP2C9*3*-F | CCACATGCCCTACACAGATGCTGT |
| *CYP2C9*3*-R | TGAATTTAATGTCACAGGTCACTGCATGGG |
| *CYP2C9*3*-UP | GTGCACGAGGTCCAGAGATACT |
| *CYP2C9*3*-DP-W | *CGCGCCGAGG* **A**TTGACCTTCTCCCCA-C_3_ |
| *CYP2C9*3*-DP-M | *CGCGCCGAGG* **C**TTGACCTTCTCCCCA-C_3_ |
| Hairpin probe | *GTCTTGTGGTACTGC* ACTCGTCTCGGTTTTCCGAGACGAGT  CCTCGGCGCGATCGTGATGAACCAT-C3 |
| AuNP-1 | SH-AAAAAAAAAAATGGTTCATCACGAT |
| AuNP-2 | GCAGTACCACAAGACAAAAAAAAAA-SH |

Notes: F, forward PCR primer; R, reverse PCR primer; UP, upstream probe; DP, downstream probe; W, downstream probe of wild-type; M, downstream probe of mutant-type; italic letters indicate the Flap fragment of either downstream probes or hairpin probe. Single bold indicate SNP sites.

| Sample | loci | genotype | Pyrosequencing map |
| --- | --- | --- | --- |
| A1 | *CYP2C19*2* | heterozygote | 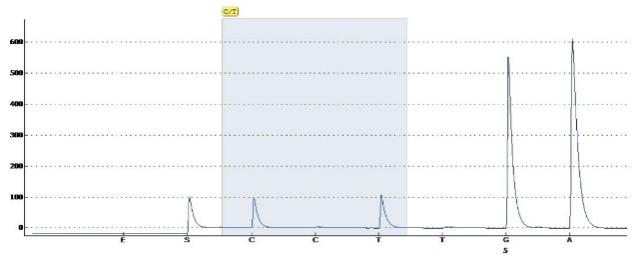 |
|  | *CYP2C19*3* | wild homozygote | 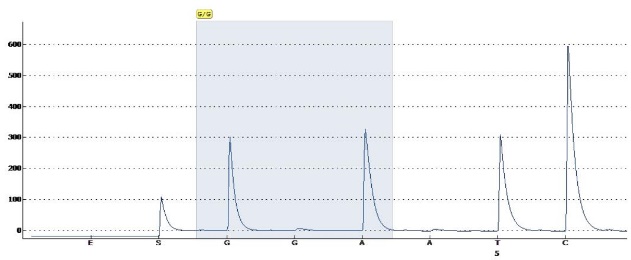 |
| A2 | *CYP2C19*2* | wild homozygote | 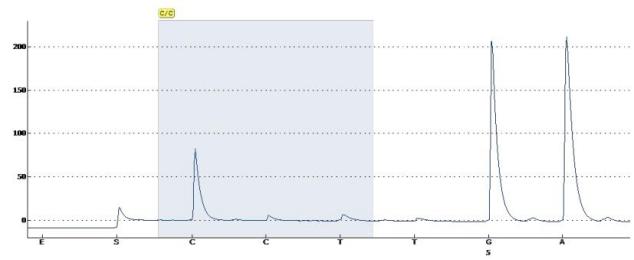 |
|  | *CYP2C19*3* | wild homozygote | 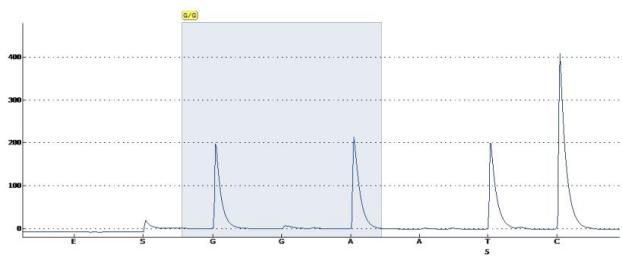 |
| A3 | *CYP2C19*2* | wild homozygote | 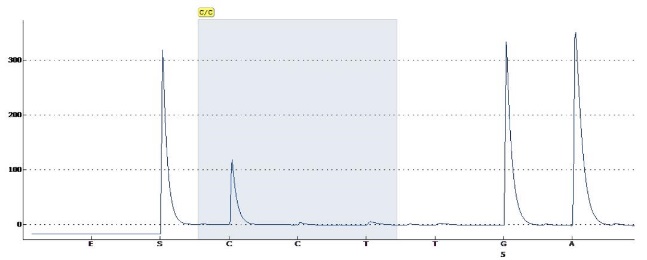 |
|  | *CYP2C19*3* | mutant homozygote | 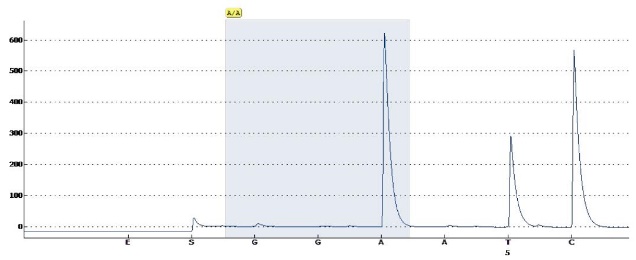 |
| A4 | *CYP2C19*2* | wild homozygote | 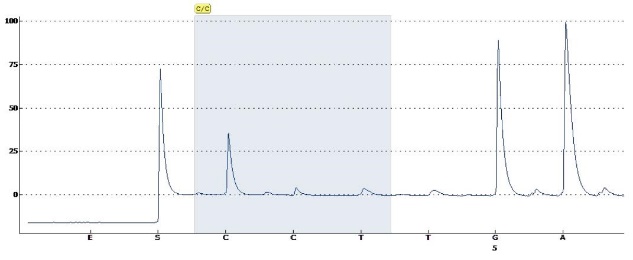 |
|  | *CYP2C19*3* | wild homozygote | 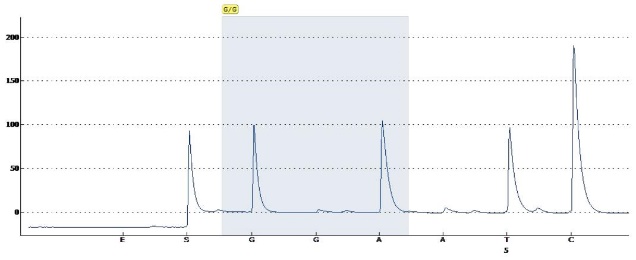 |
| A5 | *CYP2C19*2* | wild homozygote | 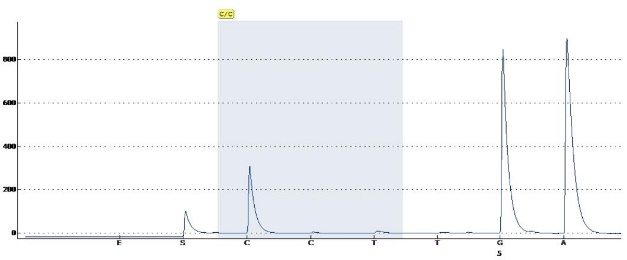 |
|  | *CYP2C19*3* | wild homozygote | 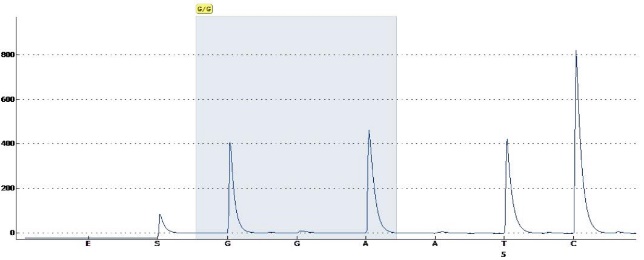 |
| A6 | *CYP2C19*2* | heterozygote | 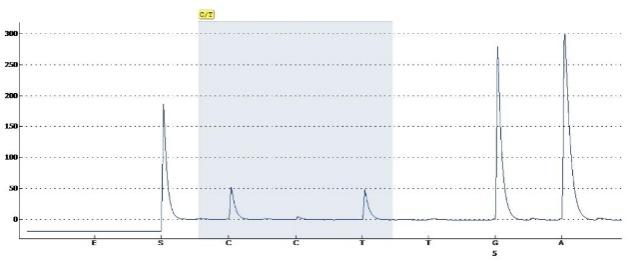 |
|  | *CYP2C19*3* | wild homozygote | 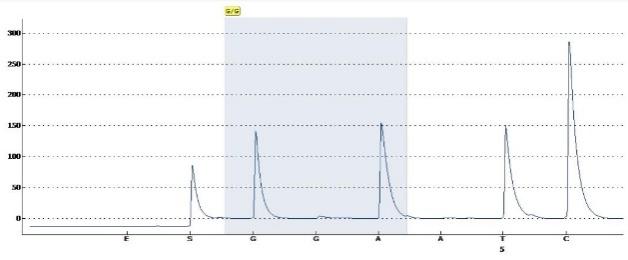 |
| A7 | *CYP2C19*2* | wild homozygote | 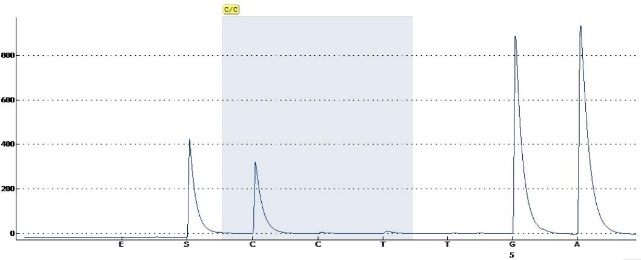 |
|  | *CYP2C19*3* | wild homozygote | 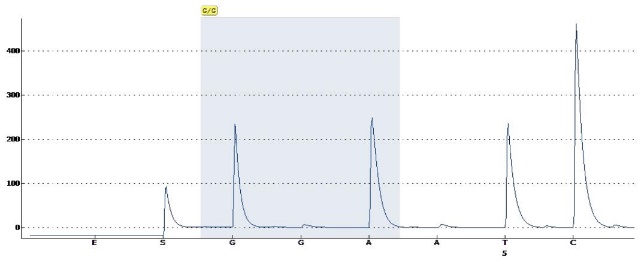 |
| A8 | *CYP2C19*2* | wild homozygote | 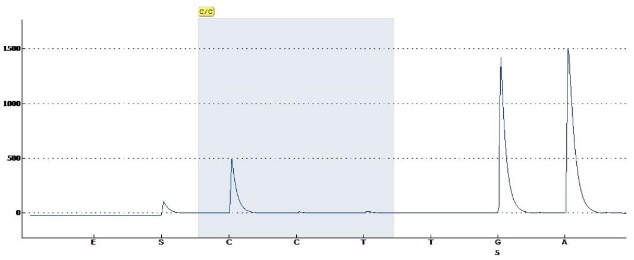 |
|  | *CYP2C19*3* | wild homozygote | 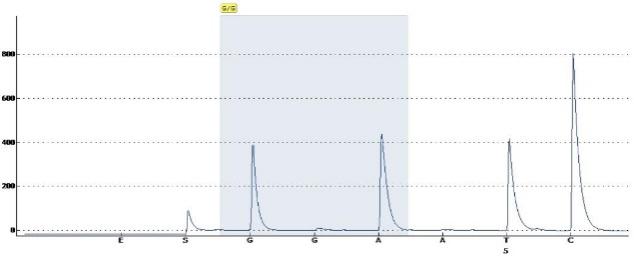 |
| A9 | *CYP2C19*2* | wild homozygote | 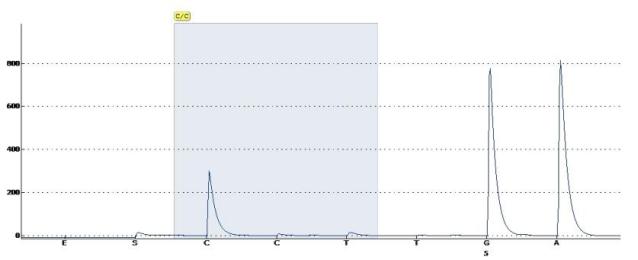 |
|  | *CYP2C19*3* | heterozygote | 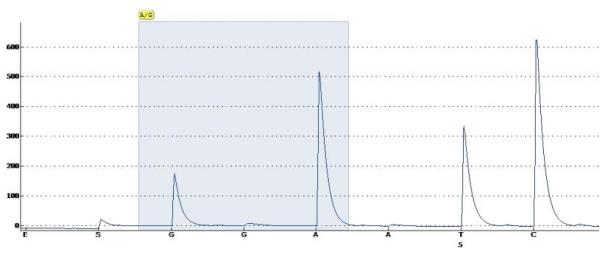 |
| A10 | *CYP2C19*2* | heterozygote | 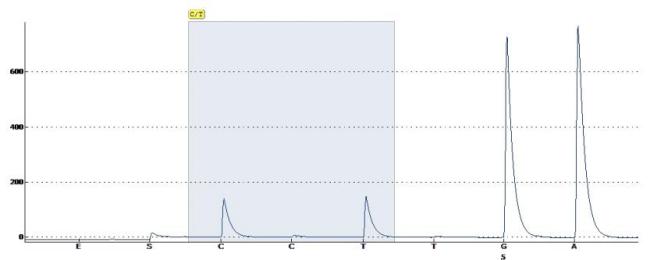 |
|  | *CYP2C19*3* | wild homozygote | 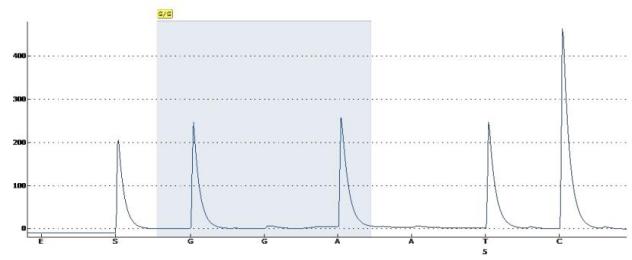 |
| A11 | *CYP2C19*2* | heterozygote | 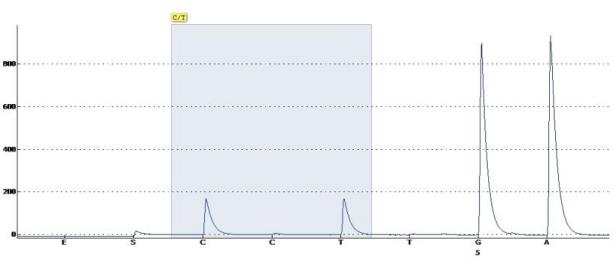 |
|  | *CYP2C19*3* | wild homozygote | 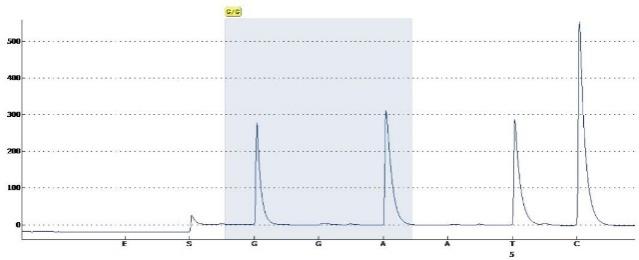 |
| A12 | *CYP2C19*2* | wild homozygote | 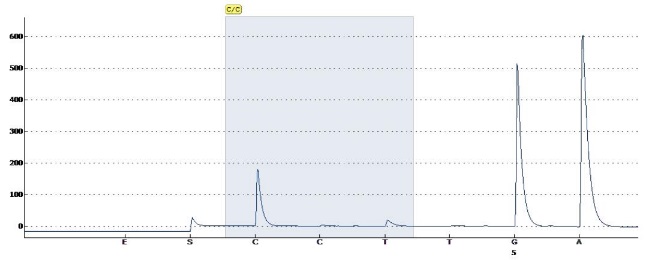 |
|  | *CYP2C19*3* | wild homozygote | 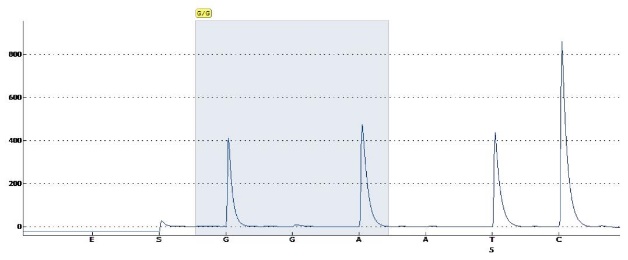 |
| A13 | *CYP2C19*2* | wild homozygote | 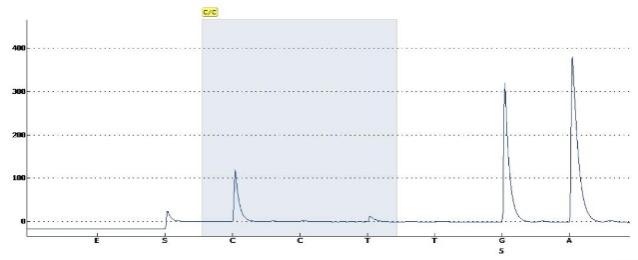 |
|  | *CYP2C19*3* | wild homozygote | 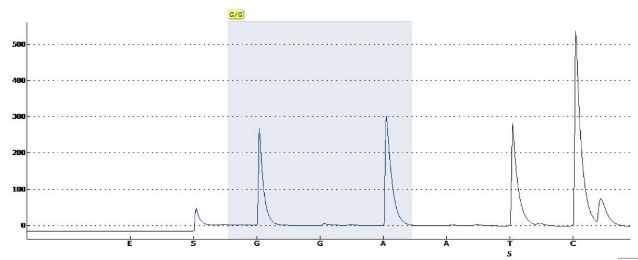 |
| A14 | *CYP2C19*2* | wild homozygote | 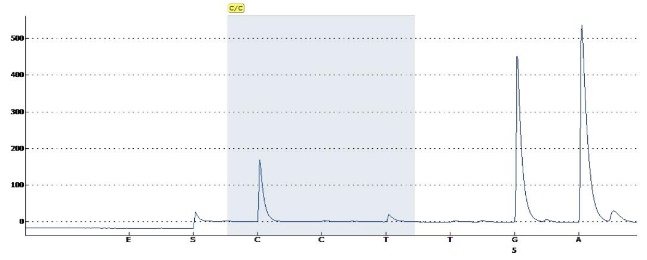 |
|  | *CYP2C19*3* | wild homozygote | 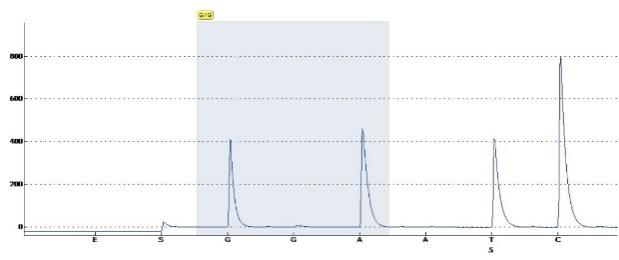 |
| A15 | *CYP2C19*2* | wild homozygote | 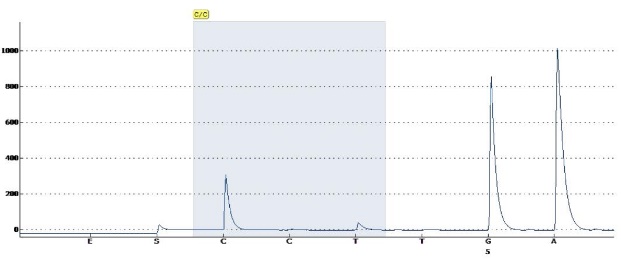 |
|  | *CYP2C19*3* | wild homozygote | 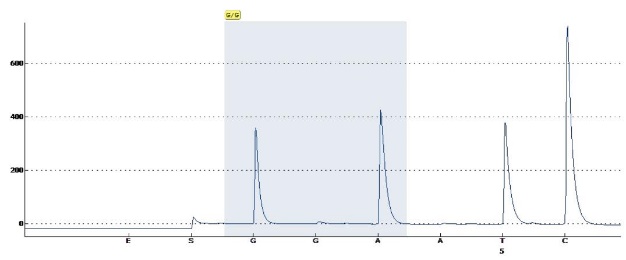 |
| A16 | *CYP2C19*2* | wild homozygote | 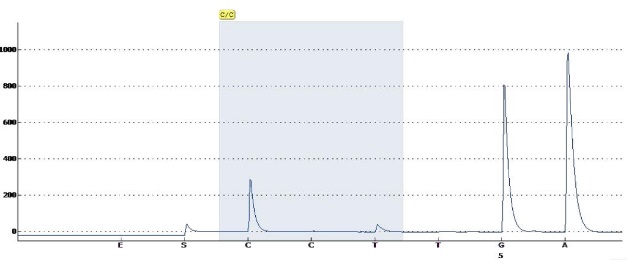 |
|  | *CYP2C19*3* | wild homozygote | 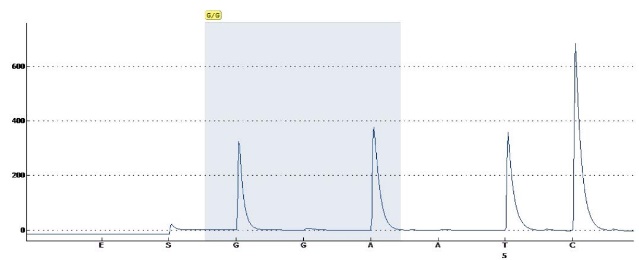 |
| A17 | *CYP2C19*2* | heterozygote | 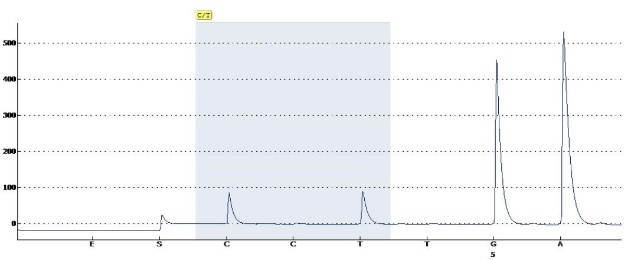 |
|  | *CYP2C19*3* | wild homozygote | 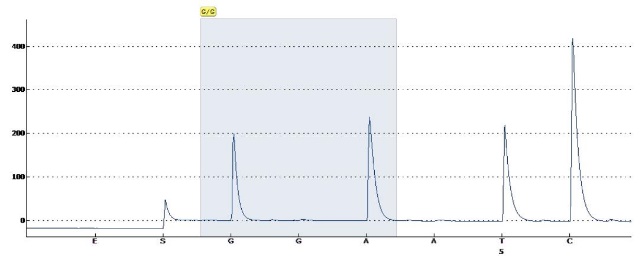 |
| A18 | *CYP2C19*2* | mutant homozygote | 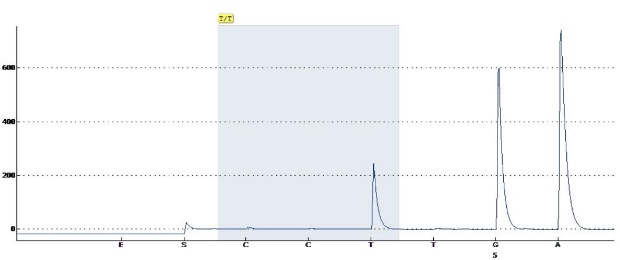 |
|  | *CYP2C19*3* | wild homozygote | 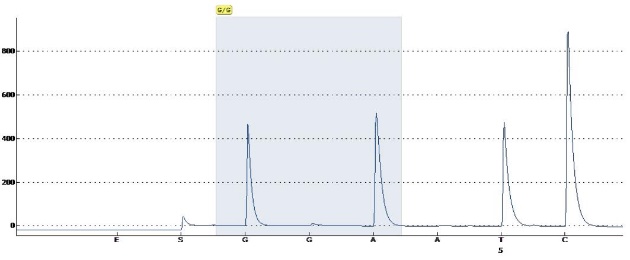 |
| A19 | *CYP2C19*2* | wild homozygote | 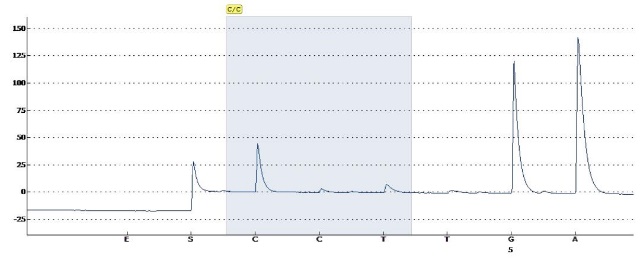 |
|  | *CYP2C19*3* | wild homozygote | 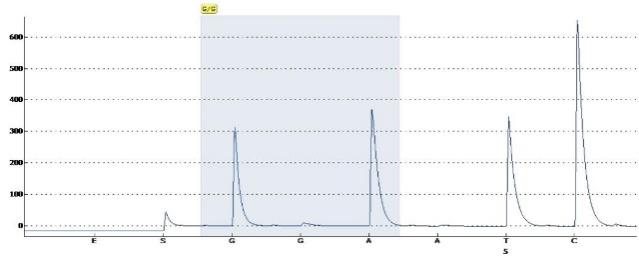 |
| A20 | *CYP2C19*2* | mutant homozygote | 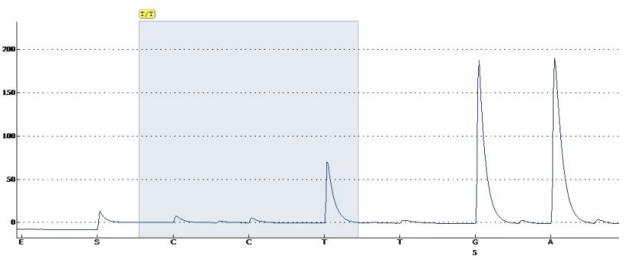 |
|  | *CYP2C19*3* | wild homozygote | 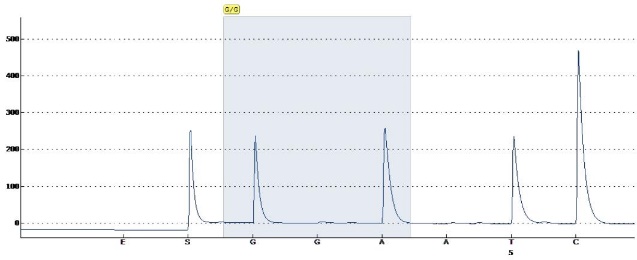 |

Supporting Table 2. Pyrosequencing map of Samples.

Note: Sequence to analyze for *CYP2C19*2:* **C/T**GGGAAATAATCAATGATAGTGGGAAAATTATTGCATATCTAAGAGAAAACAATAATTTATTAAATTAAAAGCATTTAATAAAGGTATAGATACAATATGCCAAGCTCTGG.

Sequence to analyze for *CYP2C19*3*: **G/A**ATCCAGGTAAGGCCAAGTTTTTTGCT.
